# Workshops: A Great Way to Enhance and Supplement a Degree

**DOI:** 10.1371/journal.pcbi.1003497

**Published:** 2014-02-27

**Authors:** Segun Fatumo, Sayane Shome, Geoff Macintyre

**Affiliations:** 1 H3Africa Bioinformatics Network (H3ABioNet) Node, National Biotechnology Development Agency (NABDA), Federal Ministry of Science and Technology (FMST), Abuja, Nigeria; 2 International Health Research Group, Dept of Public Health and Primary Care, University of Cambridge, Cambridge, United Kingdom; 3 Genetic Epidemiology Group, Wellcome Trust Sanger Institute, Hinxton, Cambridge, United Kingdom; 4 School of Biosciences and Technology, Vellore Institute of Technology, Vellore, Tamil Nadu, India; 5 NICTA Victoria Research Laboratory, Department of Electrical and Electronic Engineering, The University of Melbourne, Victoria, Australia; 6 Department of Computing and Information Systems, The University of Melbourne, Victoria, Australia; Princeton University, United States of America

## Abstract

As part of the International Society for Computational Biology Student Council (ISCB-SC), Regional Student Groups (RSGs) have helped organise workshops in the emerging fields of bioinformatics and computational biology. Workshops are a great way for students to gain hands-on experience and rapidly acquire knowledge in advanced research topics where curriculum-based education is yet to be developed. RSG workshops have improved dissemination of knowledge of the latest bioinformatics techniques and resources among student communities and young scientists, especially in developing nations. This article highlights some of the benefits and challenges encountered while running RSG workshops. Examples cover a variety of subjects, including introductory bioinformatics and advanced bioinformatics, as well as soft skills such as networking, career development, and socializing. The collective experience condensed in this article is a useful starting point for students wishing to organise their own tailor-made workshops.

## Why Workshop?

A workshop requires participants to take a hands-on approach to immediately implement the skills they are learning. Workshops provide insight into diverse topics and motivate students to explore new areas of interest. They are generally planned to accommodate a relatively small set of participants, encouraging individual attention to each attendee by a facilitator. In the context of Regional Student Groups (RSGs), a workshop is a training course where the participants work individually and/or in a group to solve tasks related to their areas of research in order to gain hands-on experience. This form of learning is particularly appealing for students, as it can be easily tailored to address varying needs, and it complements curriculum-based learning. Workshops run by RSGs in the past have shown that the training needs of bioinformatics students across the world are diverse. For instance, in developing nations, students typically request workshops covering basic bioinformatics skills. This may be due to a lack of formal training in the area, as not many universities outside the developed world offer bioinformatics degrees. Where bioinformatics training is already available, RSGs tend to organise project- or area-specific workshops, usually designed in response to industry requirements outside the conventional undergraduate or graduate curriculum. As bioinformatics is an emerging field, specialized workshops play a significant role in filling such curricular gaps.

## Regional Student Group Workshops

RSG workshops provide practical hands-on experience in a range of analytical techniques. Their success is in part because they respond to the needs of student members, and therefore tend to be both relevant and timely. In what follows, we describe several types of workshops, using examples from both developing and developed nations. Our goal is to show how RSG workshops may address different kinds of needs among different student populations.

In 2009, RSGs in Africa co-organised a workshop at the University of Bamako in Mali. The topics covered in the workshop included “An Introduction to R”, “Microarray Analysis Using R/Bioconductor”, “Exploring the PlasmoDb Database”, “Structural Bioinformatics”, and “Sequence Analysis Using EMBOSS”. In a similar manner, RSGs in Africa also organised a workshop at the University of the Western Cape that has now become a part of the ISCB Africa African Society for Bioinformatics and Computational Biology's biannual conference. Topics covered in this workshop included “Sequence Searching and Alignment”, “Using the EMBNET eBioKit”, “An Introduction to EMBOSS”, “Performing GWAS/Population Genetics”, and “An Introduction to Galaxy”, among others. In each session, participants worked on hands-on exercises, which imparted practical knowledge about these topics beyond the level usually available to these students. In their feedback, many students noted that the exercises were tremendously involved, with the analyses they worked on being more intricate than expected and requiring expert knowledge of the tools being applied. Generally, participants felt more confident in dealing with analyses of the course data as expert facilitators guided them. We believe that this form of learning is particularly beneficial for the advancement of participants' careers, as many find that they gain greater experience than the workshop descriptions had led them to expect.

There have also been workshops where the content developed above and beyond what was originally advertised due to the dynamic nature of the workshop facilitator. For example, in 2011, RSG-India organised workshops on “Career Planning” and “Microarray Technology” at VIT University in Vellore. Dr. Kshitish Acharya, a senior scientist at Institute of Bioinformatics and Applied Biotechnology Bangalore, taught a workshop comprising two sessions: the first focussed on the technique of microarray analysis, the second, on the discussion of a career plan. The second session considered various aspects, including how to initiate a start-up business in the life sciences sector. In addition to this, there was some insightful discussion around the scope of bioinformatics in India and how this differs from its scope in other countries. These discussions provided invaluable insight for attendees, many of them undergraduate students keen to learn about future prospects in research and entrepreneurship in bioinformatics.

An RSG retreat for young scientists in bioinformatics and computational biology was organised by the RSG-Netherlands on April 23, 2012. The retreat was a pre-event preceding the Netherlands Bioinformatics Conference 2012. The retreat provided a unique opportunity to meet other young researchers in the field, have informal discussions, and initiate new collaborations. The program included a practical workshop on “Communications and Convincing Your Supervisor.” Harmen Bussemaker, associate professor at Columbia University, gave an inspiring talk about his career path and the importance of good communication.

As we can see from the above examples, applied scientific knowledge is not the only valuable outcome of a workshop; there are subtler, yet incredibly useful, benefits usually not mentioned in the canonical program for the workshop. Among these benefits are opportunities to network and get connected with colleagues, peers, and senior scientists with the same or similar research interests.

## Tips on How to Run a Successful Workshop

The main aim of a workshop can be information sharing, problem solving, and/or training. It is important to clearly define the scope of the workshop and ensure that the facilitator or facilitators are experts in the field. When planning a workshop, it is important to understand the background of the audience, as a too narrow or too simple topic means that most attendees will be bored. If the workshop is on a specific topic, make sure to list the required background knowledge in advance. A clear agenda should be arranged at the outset of the workshop, including content that addresses the requirements of the participants and is tailored to them. For example, a particular audience may be region specific. A workshop that worked well in the United States may need to be adapted to work elsewhere; in Africa, for example, a common focal point is infectious diseases, so a workshop in microarray analysis that used cancer patient samples in the U.S. might be better with samples changed to HIV patients if being offered in Africa.

Workshop organisers should be aware of the type of techniques and/or methodologies being used by their prospective audiences, then apply this knowledge to the workshop program to help assist students in these areas. Workshops that are designed to train the audience in a new or emerging area should have content relevant to the type of work that is being conducted in each region.

The frequency at which workshops are held can impact on their success. Factors to take into account in scheduling workshops can also include how much training is required for them and how practical it is to run them. Available resources, such as funding, and local demand for the topic play a crucial role in determining workshop frequency. In some cases, it makes sense to run a workshop in one complete day, whereas in others it may be more practical to split workshop sessions over two days, maybe in half-day periods, to enable additional time to practice some of the skills.

Venue choice is also important. It is good to make sure that the workshop is held in a suitable location, preferably one participants can commute to easily and with easy access to elementary resources.

## Challenges Faced in Running a Workshop

Organising a workshop takes careful planning and effort. Many of the challenges encountered in organising a workshop can be attributed to either a lack of adequate preparation or lack of funding. If the workshop requires substantial funding, it is important to create a budget at the onset of planning and secure the funds necessary to cover all expenses. Do not commit to run a workshop without first making sure budget and logistics allow it. Try to be creative in keeping costs down. If the workshop requires a computer, see if a university computer lab can be used. Most universities are open to supporting student-run workshops.

In most cases, when something goes wrong it is due to a lack of adequate preparation. While a lot of effort may be put into planning the activities for the day, a number of things can be easily overlooked; for instance, not providing enough advertising can result in an insufficient number of participants.

If the workshop venue is too far from the intended participants, accessibility can become a drawback. Although it is impossible to ensure that everybody is happy, the most practical approach is to select a destination that satisfies most conditions and explore ways to make it easier for travel (e.g., travel grants).

When working with software-based workshops, it is wise to ensure that all computers are adequately prepared in advance with all the relevant software. This avoids delays or interruptions on the day of the workshop. The same applies to internet-based workshops: connections should be set up and checked well in advance to avoid delays.

## Conclusion

Workshops have proven to be a successful activity for most Regional Student Groups in the ISCB Student Council network. Apart from enhancing the technical and analytical skills of participants, we have shown that they provide unanticipated skills not easily available through the regular curricular programs. The organization of workshops has afforded an ideal way to keep current RSG members interested in participating more fully in the group while making it more attractive for new members to join.

**About the Authors**

The authors have been involved in organising various events for Regional Student Groups and the ISCB Student Council.

**Segun Fatumo** was the founder and president of RSG-Africa (2007–2009) and is currently Vice Chair of the RSG WorldWide program (2011–present).

**Sayane Shome** is the current president of RSG-India (2012–present).

**Geoff Macintyre** served as the chair of the ISCB Student Council (2011–2012) and was the co-founder and president of RSG-Australia (2009–2010).

